# Years of life lost due to external causes in Honduras, 2013-2023

**DOI:** 10.17843/rpmesp.2026.431.15420

**Published:** 2026-03-31

**Authors:** Augusto A. Rosales-Meléndez

**Affiliations:** 1 Independent researcher; Tegucigalpa, Honduras.

**Keywords:** Life Expectancy, Mortality, External Causes, Honduras, Mortality, Premature

## Abstract

**Objectives.:**

To estimate the years of life lost (YLL) for deaths due to external causes in Honduras during the 2013-2023 period.

**Materials and methods.:**

A descriptive-ecological study based on mortality records for external causes from the National Observatory on Violence of Honduras (2013-2023). YLL were estimated, and a stratified analysis by sex, age, and type of cause was performed. Additionally, a trend analysis using the Mann-Kendall test was conducted, stratified by sex and type of cause.

**Results.:**

During the analyzed period, 1.9 million YLL due to external causes were estimated, 86% of which corresponded to men. Homicides were the main cause of YLL in both sexes (62.4% in men and 43.5% in women), followed by traffic accidents (18.3% and 25%, respectively). Between 2013 and 2023, YLL showed a steady increase. In men, increases were observed in unintentional causes (110.5%), suicide (100.1%), and traffic accidents (98.7%); in women, in unintentional causes (146.2%), suicide (62.6%), and traffic accidents (40.4%).

**Conclusions.:**

Deaths due to external causes, which are mostly preventable, disproportionately affect the young adult population and show an increasing trend. It is essential to implement interventions differentiated by age and sex that contribute to reducing their impact on premature mortality.

## INTRODUCTION

Deaths due to external causes include homicides, traffic accidents, suicides, and other unintentional injuries such as falls, electrocution, and occupational accidents; they represent one of the main causes of mortality in both high-income ^1^ and low-income countries ^2^. In the region of the Americas alone, the loss of 38 million years of life lost (YLL) due to deaths from external causes was estimated for the year 2019 ^3^.

In the case of Honduras, by 2021, deaths from external causes represented the second leading cause of disease burden after cardiovascular diseases ^4^. These deaths disproportionately affect men and young people of productive age, generating significant losses not only in terms of health but also in human capital, productivity, and family well-being.

Taking into account the magnitude of deaths from external causes, it is essential not only to describe their frequency but also to perform an adequate epidemiological stratification by sex, age group, and type of external cause. This approach allows for a more precise visualization of the most affected groups and guides the design of specific public policies focused on age and sex. In this context, the use of more advanced disease burden metrics such as YLL, and their disaggregation by sex, age, and cause, provides critical information for decision-making in public health. Therefore, the objective of this study was to estimate the disease burden measured in YLL for deaths from external causes in Honduras during the 2013-2023 period.

**KEY MESSAGES**

Motivation for the study. Deaths due to external causes generate high mortality in Honduras; however, their impact is rarely quantified using years of life lost (YLL).

Main findings. Between 2013 and 2023, 1.9 million years of life were lost due to external causes. In both sexes, the main causes were homicides, traffic accidents, suicides, and unintentional deaths. The greatest burden was concentrated in adults under 30 years of age.

Implications. Interventions differentiated by age and sex are required to prevent homicidal violence, traffic accidents, and suicides.

## MATERIALS AND METHODS

### Study design

A descriptive-ecological study was conducted through secondary data analysis of deaths from external causes in Honduras during the 2013-2023 period. The information was obtained from the National Observatory on Violence (ONV), an organization that collects and consolidates mortality records from the National Police (PN), the Directorate of Forensic Medicine (DMF), and the National Registry of Persons (RNP), by downloading open and anonymized databases ^5^. Additionally, to perform a more detailed statistical analysis, access to disaggregated records was requested from the ONV via email.

### Definition of variables

The classification of external causes was performed by the ONV and was used according to the categories recorded by that institution. Sex was categorized as female and male. External causes were classified into homicide, suicide, traffic accidents, unintentional causes (falls, burns, electrocution, poisonings, burials/landslides, and explosions), and non-consigned external causes, corresponding to undetermined mechanisms of death. Age was stratified into five-year intervals to maintain consistency with methodological requirements for age standardization and as a requirement for calculating YLL.

### Statistical analysis

The Global Burden of Disease (GBD) ^6^ methodology was followed for the calculation of YLL. First, the standard life expectancy was calculated using the UN life tables ^7^ for Latin America; corresponding values were assigned by sex for life expectancy at birth, using World Bank estimates as a reference ^8^. Subsequently, for each age group, standard life expectancy was interpolated based on the average age of death. The calculation of standard life expectancy was performed using Equation 1 ^6^ (supplementary material).

Subsequently, YLL were calculated by multiplying the number of deaths, stratified by age, sex, and external cause, by the years of life expected according to the standard life expectancy. For each age group, the average age of death and the corresponding interpolated standard life expectancy were used, via Equation 2 (supplementary material).

The parameters were conservative according to GBD recommendations ^6^, which include: not applying age discounting, using an age-weighting constant of 0.04, and an age adjustment of 0.165.

Other derived measures were also estimated, such as average YLL, calculated as the total YLL divided by the number of deaths, stratified by sex, age, and cause. Complementarily, the YLL ratio between men and women was estimated, using men as the reference category.

For the trend analysis, the Mann-Kendall test was used along with the associated p-value and the slope estimated by Sen's method. Additionally, a Joinpoint regression was performed to identify possible change points in YLL trends, considering the COVID-19 pandemic period (2020-2023) as an event of interest. For this analysis, age-standardized YLL rates per 100,000 inhabitants were calculated, using population projections from the National Institute of Statistics of Honduras (INE) and the World Health Organization (WHO) world standard population as weights. Joinpoint regression was performed using the Joinpoint Trend Analysis Program software, version 5.4.0. The results were presented using trend graphs and tables of absolute and relative frequencies. Statistical analyses were performed in RStudio (version 2024.12.1; RStudio Team, Boston, MA, USA), using the “dplyr” and “tidyr” packages for data organization, the “yll” package for YLL calculation, and the “trend” package for trend analysis.

### Ethical considerations

This study was conducted in accordance with the ethical principles established in the Declaration of Helsinki for medical research. Confidentiality was guaranteed through the exclusive use of completely anonymized secondary data. Since no identifiable information was included, no contact with individuals was involved, and it is a secondary analysis of open data, approval from an ethics committee was not required, in accordance with the International Ethical Guidelines for Epidemiological Studies ^9^.

## RESULTS

During the 2013-2023 period, 74,724 deaths from external causes were recorded in men, of which 1,753 (2.3%) were excluded due to lack of information on age. In women, 11,095 deaths were reported, with 190 (1.7%) being excluded for the same reason. With the available data, a total loss of 1.9 million YLL was estimated in Honduras, of which 86% (1.64 million) corresponded to men (Table 1).

**Table 1:** Years of life lost (YLL) by sex and cause, Honduras 2013-2023.

Causes	Women				Men				
	Deaths	YLL	%	Average YLL	Deaths	YLL	%	Average YLL	Ratio* M/W
Traffic accidents	2695	61845.5	24.1	22.9	13823	300353.8	18.3	21.7	5:1
Homicides	4586	111682.0	43.5	24.3	43640	1024040.7	62.4	23.5	9:1
Non-consigned	1467	34359.0	13.4	23.5	5256	105775.4	6.4	20.1	3:1
Unintentional	1233	24920.5	9.7	20.2	6531	134689.1	8.2	20.6	5:1
Suicide	924	23737.4	9.3	25.8	3541	77516.2	4.7	21.9	3:1
Total	10905	256544.4	100	22.5	72791	1642375.2	100	23.5	6:1

* Men: Women YLL Ratio.

In both sexes, the main cause of YLL was homicide. In men, 1 million YLL were lost (62.4%), while in women, it was 111,000 YLL (43.5%), with a male:female YLL ratio of 9:1. The second cause was traffic accidents, with 300,000 YLL in men (18.3%) and 61,000 in women (25%) (Table 1)

In other causes such as unintentional deaths, suicide, and non-consigned external causes, equally high male: female ratios were observed: 5:1, 6:1, and 3:1, respectively. It is noteworthy that, in relative terms, women recorded 34,000 YLL from unspecified external causes (13.4%), while men accumulated 100,000 YLL (6.4%). In the case of suicide, women lost 23,000 YLL (9.3%) and men 70,000 YLL (4.7%) (Table 1). 

During the 2013-2023 period, trend analysis using the Mann-Kendall test and Sen’s slope showed statistically significant increasing trends in YLL due to unintentional causes, suicide, and traffic accidents in both sexes. The only exception was traffic accidents in women, for which no statistically significant trend was identified. In contrast, homicides presented decreasing trends in both men and women (Table 2).

**Table 2:** Temporal trends and slopes due to external causes in Honduras 2013-2023.

External cause	Men			Women		
	Tau^a^	p-value^b^	Slope^c^	Tau^a^	p-value^b^	Slope^c^
Unintentional	0.78	0.001	544 (326.2 a 900.8)	0.75	0.001	131 (39.4 a 236.1)
Suicide	0.85	<0.001	559 (457.5 a 721.8)	0.60	0.01	104 (45.1 a 179.3)
Traffic	0.81	<0.001	1810 (1331.8 a 2324.6)	0.27	0.27	75 (-75.6 a 262.0)
Homicide	-0.81	<0.001	−6290 (-9172.3 a -2807.4)	-0.75	0.001	-648 (-892.7 a -364.7)

a Mann-Kendall Tau, ^b^ Mann-Kendall p-value, ^c^ Slope value in years of life lost (Sen's method).

**Figure 1:**
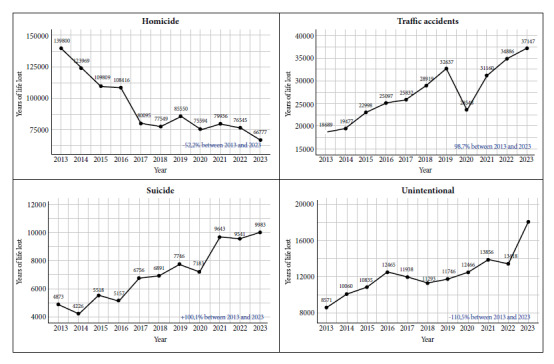
Years of life lost due to external causes in men, Honduras, 2013-2023.

**Figure 2:**
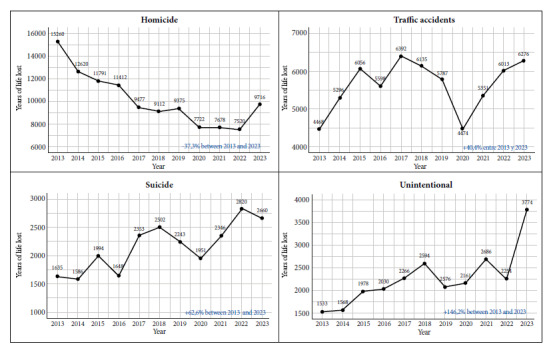
Years of life lost due to external causes in women, Honduras, 2013-2023.

From 2013 to 2023, YLL in men increased by 110.5% for unintentional causes, 100.1% for suicides, and 98.7% for traffic accidents, while homicides decreased by 52.2% (Figure 1). A similar pattern was observed in women, with increases of 146.2% in unintentional causes, 62.6% in suicides, and 40.4% in traffic accidents, and a 37.3% reduction in YLL due to homicide (Figure 2). In both sexes, a decrease in YLL due to traffic accidents was observed in the year 2020.

In men, those under 30 years of age concentrated 51.0% of the accumulated YLL from traffic accidents, 53.9% from homicides, 49.3% from suicides, and 44.6% from unintentional causes (Table 3). Likewise, when considering women under 30 years of age, they accumulated 53.6% of YLL from traffic accidents, 54.7% from homicides, 73.9% from suicides, and 61.6% from unintentional causes. The 15 to 19 age group stands out, representing 31% of YLL due to suicide in women, as well as the 25 to 29 age group, which contributed the largest individual percentage for the four causes described (Table 4). The Joinpoint regression trend analysis did not identify statistically significant change points in YLL trends for any of the types of external causes analyzed in both sexes, indicating the presence of a single temporal trend throughout the study period, including the years of the COVID-19 pandemic (supplementary material).

**Table 3:** Years of life lost by age and external cause in men, Honduras, 2013-2023.

Age	Traffic accidents n (%)	Cumulative percentage ^a^	Homicides n (%)	Cumulative percentage ^a^	Suicide n (%)	Cumulative percentage ^a^	Non- consigned n (%)	Cumulative percentage ^a^	Unintentional n (%)	Cumulative percentage ^a^
0-4	5272 (1.8)	1.8	5298.7 (0.5)	0.5	-	-	14737.1 (13.9)	13.9	8421.1 (6.3)	6.3
5-9	6601.2 (2.2)	4.0	2191.2 (0.2)	0.7	143 (0.2)	0.2	1150.4 (1.1)	15	5101 (3.8)	10.1
10-14	8233.5 (2.7)	6.7	9926.3 (1)	1.7	1850.6 (2.4)	2.6	1659.2 (1.6)	16.6	5622.1 (4.2)	14.3
15-19	28624.7 (9.5)	16.2	116563.7 (11.3)	13.0	8672.6 (11.2)	13.8	4312.2 (4.1)	20.7	12180.4 (9)	23.3
20-24	56260.5 (18.7)	35.0	216129.2 (21.1)	34.1	14721.9 (19)	32.8	7944.7 (7.5)	28.2	14075.1 (10.5)	33.8
25-29	48109.4 (16)	51.0	202710.1 (19.8)	53.9	12851.7 (16.5)	49.3	9376.9 (8.9)	37.1	14530 (10.8)	44.6
30-34	36999,8 (12,3)	63.4	154508.4 (15.1)	69.0	8864.1 (11.3)	60.6	12100 (11.3)	48.4	13456.5 (10)	54.6
35-39	28804.7 (9.7)	73.0	112568.5 (11)	80.0	8101.5 (10.5)	71.1	10766.1 (10.2)	58.6	13100.9 (9.7)	64.3
40-44	19717.2 (6.6)	79.6	76630.5 (7.5)	87.5	6839.3 (8.8)	79.9	9394.9 (8.9)	67.5	11442.3 (8.5)	72.8
45-49	15944.1 (5.3)	84.9	50734.5 (5)	92.5	5079.2 (6.6)	86.5	8643.5 (8.2)	75.7	9340.2 (6.9)	79.7
50-54	13357.9 (4.4)	89.3	31819.2 (3.1)	95.6	3221.4 (4.2)	90.7	6948.2 (6.6)	82.3	7948.5 (5.9)	85.6
55-59	10359.1 (3.4)	92.7	21897.9 (2.1)	97.7	2473.9 (3.2)	93.9	6502.8 (6.1)	88.4	5826.9 (4.3)	89.9
60-64	8118 (2.7)	95.4	12047 (1.2)	98.9	1797.6 (2.3)	96.2	4133.0 (3.9)	92.3	4904.1 (3.6)	93.5
65-69	6093.6 (2.0)	97.4	6249.8 (0.6)	99.5	1235.1 (1.6)	97.8	3644.6 (3.4)	95.7	3473.5 (2.6)	96.1
70-74	3562.5 (1.2)	98.6	2855.1 (0.3)	99.8	755.9 (1)	98.8	2180.6 (2.1)	97.8	2184 (1.6)	97.7
75-79	2207.1 (0.7)	99.3	1228.5 (0.1)	99.9	509.5 (0.7)	99.5	1346.4 (1.3)	99.1	1397.7 (1)	98.7
80-84	2088.6 (0.7)	100.0	682 (0.1)	100.0	399 (0.5)	100	934.7 (0.9)	100	1684.8 (1.3)	100
Total	300353.8 (100)	-	1024040.7	-	77516.2 (100)	-	105775.4	-	134689.1 (100)	-

a Cumulative percentage by cause and age.

Note: Values in parentheses represent the percentage relative to the total years of life lost by cause and age.

**Table 4:** Years of life lost by age and external cause in women, Honduras, 2013-2023.

Age	Traffic accidents n (%)	Cumulative percentage^a^	Homicides n (%)	Cumulative percentage^a^	Suicide n (%)	Cumulative percentage^a^	Not recorded n (%)	Cumulative percentage^a^	Unintentional n (%)	Cumulative percentage^a^
0-4	4490.3 (7.3)	7.3	3390.2 (3.0)	3.0	-	-	10681.1 (30.9)	30.9	6188.3 (24.8)	24.8
5-9	4061.7 (6.6)	13.8	969.5 (0.9)	3.9	29.3 (0.1)	0.1	1000.4 (2.9)	2.9	2442.1 (9.8)	34.6
10-14	3510.3 (5.7)	19.5	3930.4 (3.5)	7.4	2527.6 (10.5)	10.6	1839.1 (5.3)	5.3	2074.5 (8.3)	42.9
15-19	6232.3 (10.1)	29.6	16225 (14.5)	21.9	7371 (31.1)	41.7	2749.4 (8.0)	8.0	1686.7 (6.8)	49.7
20-24	7998.7 (12.9)	42.5	18821 (16.8)	38.7	4503.3 (19.0)	60.7	2892.6 (8.4)	8.4	1583.3 (6.4)	56.1
25-29	6862.3 (11.1)	53.6	17855.1 (16)	54.7	3139 (13.2)	73.9	2424.4 (7)	7.0	1368.7 (5.5)	61.6
30-34	6392.4 (10.3)	63.9	13484.6 (12.1)	66.8	1466.6 (6.2)	80.1	2369.2 (6.9)	6.9	1214.6 (4.9)	66.5
35-39	4819 (7.8	71.7	11714.3 (10.5)	77.3	1490.6 (6.3)	86.4	2501.3 (7.2)	7.2	983.7 (3.9)	70.4
40-44	3696.8 (6.0)	77.7	8448.6 (7.6)	84.9	1083.7 (4.6)	91.0	1970.8 (5.7)	5.7	924.9 (3.7)	74.1
45-49	3229.7 (5.2)	82.9	6386.7 (5.7)	90.6	800.5 (3.4)	94.4	1318.8 (3.8)	3.8	1179.5 (4.7)	78.8
50-54	2801 (4.5)	87.5	4329.8 (3.9)	94.5	501.4 (2.1)	96.5	1042.7 (3)	3.0	814.8 (3.3)	82.1
55-59	2287 (3.7)	91.2	2560.9 (2.3)	96.8	435.8 (1.8)	98.3	1150.3 (3.3)	3.3	482.8 (1.9)	84.0
60-64	2004.8 (3.2)	94.4	1656.7 (1.5)	98.3	151 (0.6)	98.9	591.8 (1.7)	1.7	762.3 (3.1)	87.1
65-69	1603.7 (2.6)	97.0	879.9 (0.8)	99.1	93.9 (0.4)	99.3	745.5 (2.2)	2.2	493.9 (2.0)	89.1
70-74	879.8 (1.4)	98.4	481.9 (0.4)	99.5	75.9 (0.3)	99.6	505.2 (1.5)	1.5	639.1 (2.6)	91.7
75-79	508.6 (0.8)	99.2	314.1 (0.3)	99.8	61.5 (0.3)	99.9	298.1 (0.9)	0.9	631.4 (2.5)	94.2
80-84	467.2 (0.8)	100.0	233.2 (0.2)	100.0	6.2 (0.1)	100	458.9 (1.3)	1.3	1450 (5.8)	100
Total	61845.5 (100)	-	111682 (100)	-	23737.4 (100)	-	34539.6 (100)	100.0	24920.5 (100)	-

a Cumulative percentage by cause and age.

Note: Values in parentheses represent the percentage of the total years of life lost by cause and age.

## DISCUSSION

Between 2013 and 2023, the evolution of YLL due to external causes in Honduras showed changes in the mortality profile. Although homicides continued to be the predominant cause, their relative weight decreased considerably in both sexes. In contrast, YLL associated with traffic accidents, suicide, and unintentional causes experienced sustained growth. This pattern suggests progress in controlling homicidal violence but also highlights an increase in other deaths from external causes that require urgent public health attention.

Beyond the changes in the profile of mortality from external causes, a marked asymmetry by sex persists in homicide mortality, with a consistent predominance of men across all age groups. This pattern has been widely documented both in countries close culturally and geographically, such as Mexico ^10^, and in more distant contexts, such as Iran ^11^.

One possible explanation for the sex inequality has been attributed to higher male participation both as perpetrators and victims of violence. It is estimated that approximately 9 out of 10 violent crimes are committed by men ^12^ and 81% of victims are also men ^13^. This dual condition has been linked to structural factors such as participation in organized crime networks and exposure to interpersonal violence, especially among youth aged 15 to 29 ^13^. A greater tendency among men to resolve conflicts through the use of force has also been noted ^14^, as well as the influence of masculinity norms that reinforce violence as a form of social validation ^15^. These data support the need for policies targeted at young men at risk.

Traffic accidents constituted the second leading cause of YLL, with a higher absolute burden in men. These sex differences are consistent with previous studies. In line with this, it has been reported in Iran that men present more than triple the YLL from this cause compared to women ^16^, while in Mexico, a male predominance has also been observed across all ages in mortality from traffic accidents ^17^.

From an etiological approach, these sex differences in the YLL burden from traffic accidents can be explained, in part, by factors such as the male predominance in the role of motorcycle driver, a means of transport associated with high lethality ^18^. Likewise, a greater tendency among men to engage in reckless behavior and to fail to comply with traffic regulations has been described, both as drivers ^19^ and pedestrians ^20^. The increase in YLL from traffic accidents during the 2013-2023 period could be associated with the growth of the vehicle fleet, increased road density, and a higher risk of accidents in the urban environment ^21^. In 2020, a point interruption in the trend was recorded, with a decrease in YLL in both sexes, probably associated with the isolation measures implemented during the COVID-19 pandemic and the resulting reduction in vehicle mobility; a similar behavior in mortality was reported in other countries that adopted analogous containment measures ^22^.

Regarding suicide, the YLL analysis also found a marked predominance in men; however, the relative burden was twice as high in women (9.3% versus 4.7%), mainly influenced by the high frequency of suicides at earlier ages. In this study, one in three YLL from suicide in women corresponded to the 15 to 19 age group. This pattern is consistent with international epidemiological series reporting a high incidence of suicide in young women ^23^. The increase in YLL associated with suicide could be related to the presence of untreated mental disorders, vulnerable sociodemographic conditions, and exposure to adverse events throughout life ^24^. These results underscore the need to strengthen mental health programs, with special emphasis on young women and men of productive age.

In parallel, YLL due to unintentional causes (such as falls, electrocution, and occupational accidents) presented a marked predominance in men, who recorded five times more YLL from this cause compared to women. This pattern has been widely documented in the literature and is presented consistently across all age groups, equalizing only at advanced ages, from 80 years onwards ^25^. This inequality can be attributed to a greater exposure of men to occupational risks that increase the probability of fatal accidents, such as physically demanding jobs or those developed under adverse environmental conditions (extreme heat, humidity, exposure to gases or toxic substances). In contrast, women tend to be more exposed to forms of psychological violence in the workplace ^26^. Furthermore, the increase in YLL from unintentional causes could be linked to both an improvement in reporting systems and precarious working conditions, non-compliance with occupational safety measures, and the extension of working days ^27^.

Regarding age distribution, those under 30 years of age represented the largest contribution to total YLL in both sexes. From a macroeconomic perspective, high mortality in this age group generates economic consequences such as a reduction in the workforce and income losses. It is estimated that, due to traffic accidents alone, the region of the Americas loses between 3% and 5% of its gross domestic product (GDP) each year, reflecting the magnitude of this avoidable burden on economic and social development (28).

This study is not without limitations. Among them is the exclusion of approximately 2% of the records due to lack of information on age, which prevented the corresponding YLL calculation. Likewise, there is the possibility of bias due to errors in data quality, cause classification, or underreporting of deaths. The absence of socioeconomic or contextual variables, as well as the deferred effects of phenomena such as the COVID-19 pandemic, which could have influenced mortality, must also be considered.

Nevertheless, the strengths include a representative sample of more than 10 years, which allows for a representative description of the burden of mortality from external causes in the country. Furthermore, standardized methodologies were used for the YLL calculation, which favors the comparability and consistency of the results.

In conclusion, deaths from external causes generate a high burden for the country, especially in men and young adults under 30 years of age. The accumulation of a YLL burden six times greater in men than in women reinforces the need to incorporate gender approaches into public policies, as well as to design focused interventions that contribute to reducing premature mortality in the most vulnerable groups.

While a decrease in YLL from homicide was found in both sexes, this cause remains the leading one among external deaths, underscoring the importance of maintaining and strengthening violence and crime prevention strategies. On the other hand, the increase in YLL from traffic accidents, suicide, and unintentional causes in both sexes highlights the urgency of implementing differentiated actions by age group and gender that comprehensively address road safety, mental health, and safety conditions in occupational and community environments.
