# Supplementary material for: Years of life lost due to external causes in Honduras, 2013-2023
Source: Rev Peru Med Exp Salud Publica. 2026 Mar 31;43(1):69–76. doi: 10.17843/rpmesp.2026.431.15420 (PMC13245990; doi:10.17843/rpmesp.2026.431.15420)
Supplement: Supplementary material. — Available in the electronic version of the RPMESP. [file rpmesp-43-01-15420-s001.docx]

# **MATERIAL SUPLEMENTARIO**

**Ecuación 1: cálculo de la esperanza de vida estándar**


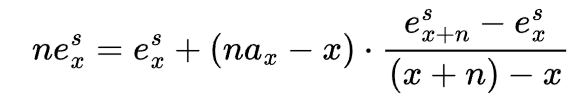


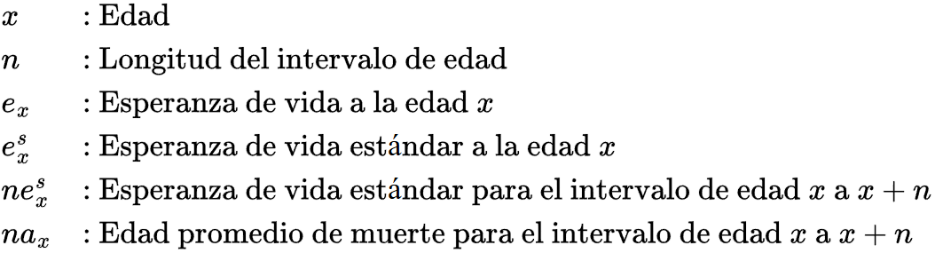
 Donde:

**Ecuación 2: cálculo de los AVP**


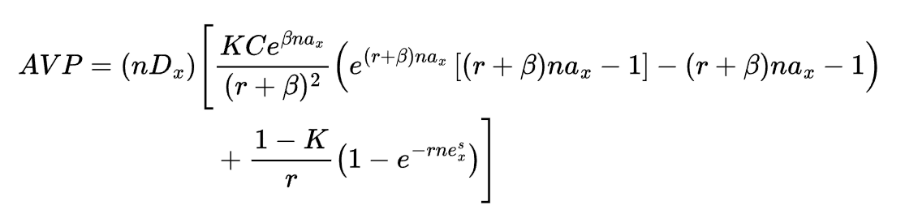


Donde:


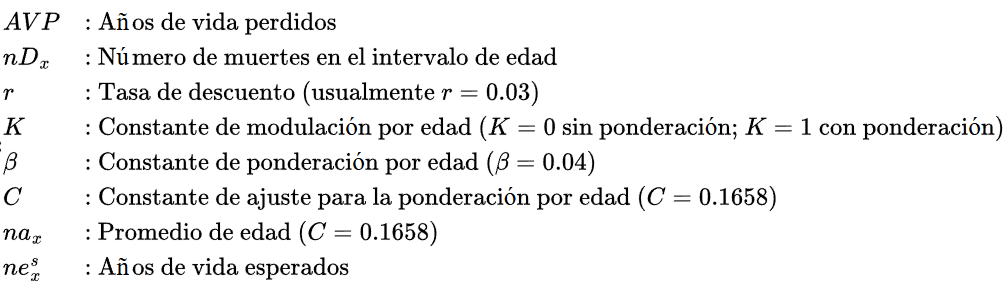


**Figura 1. Joinpoint regression de años de vida perdidos estandarizados por edad por 100,000 habitantes en causas externas en hombres, Honduras, 2013-2023**


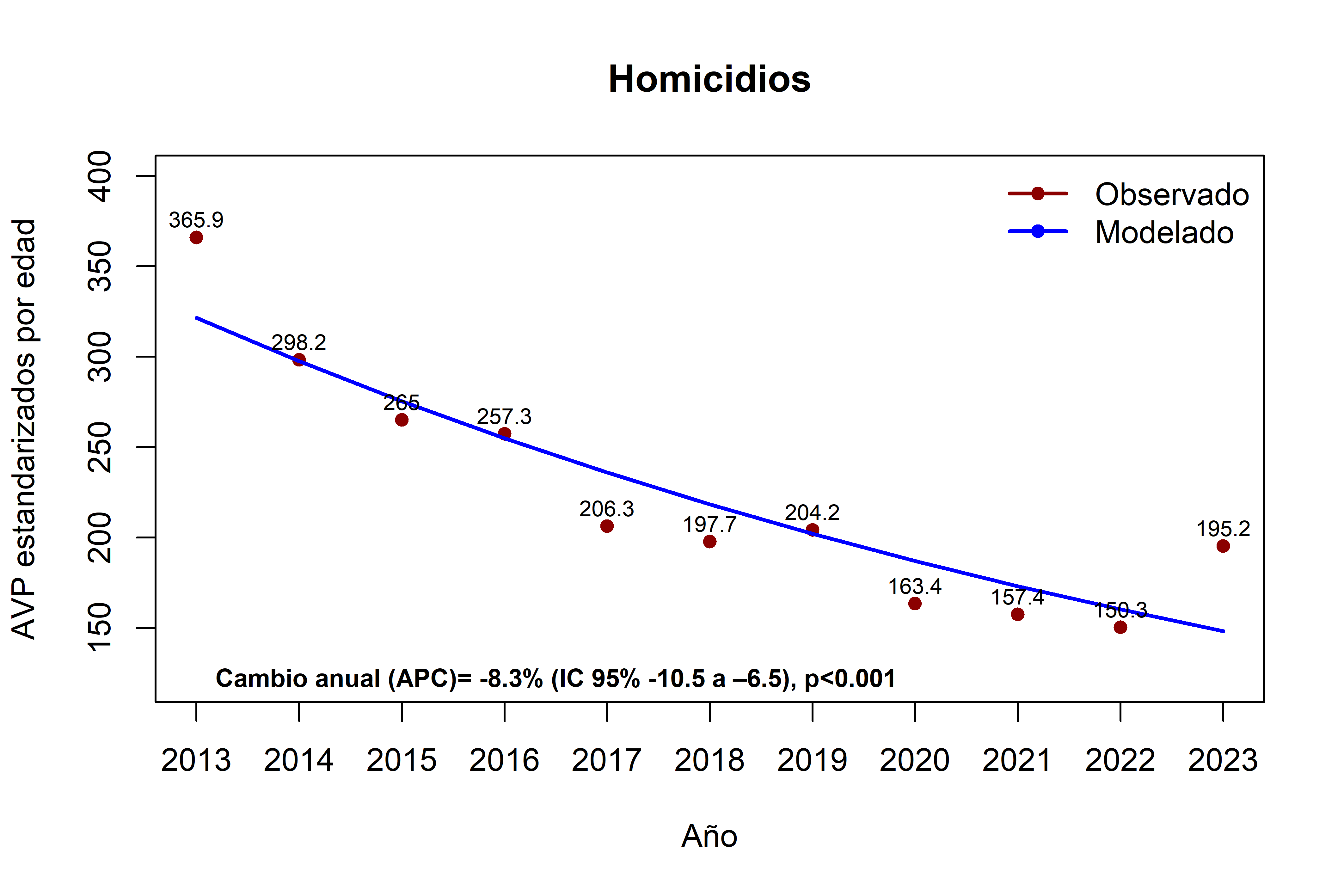

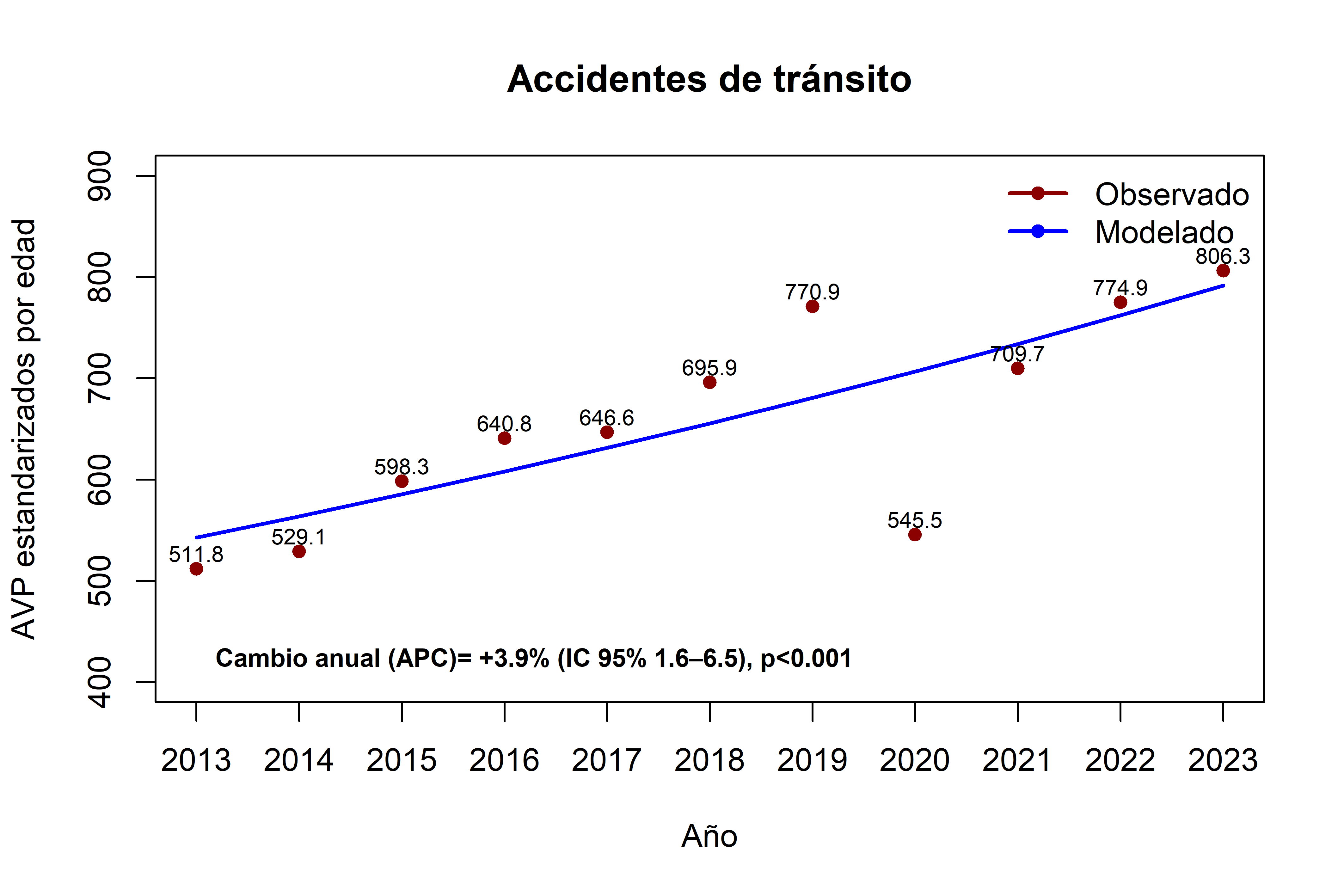

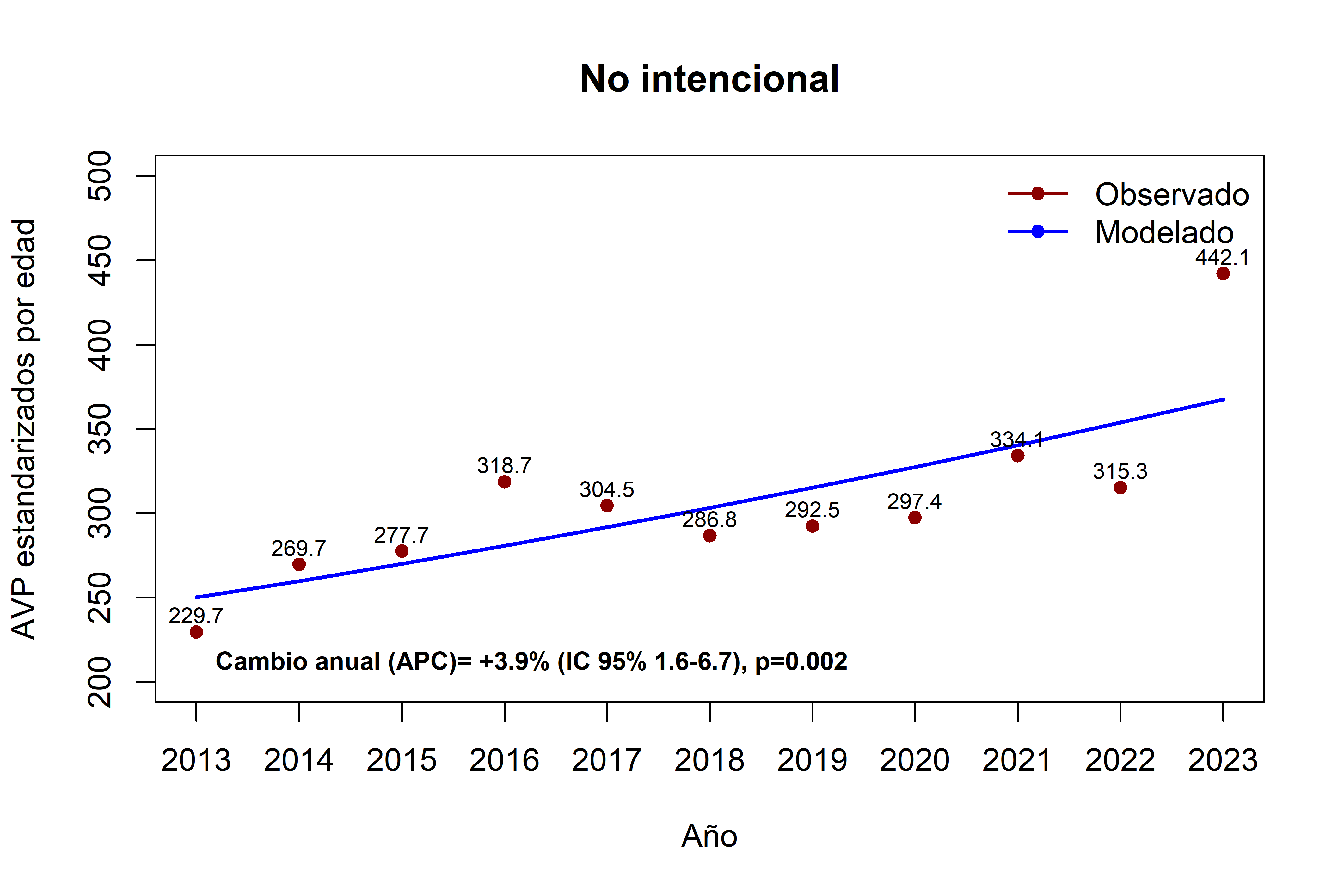

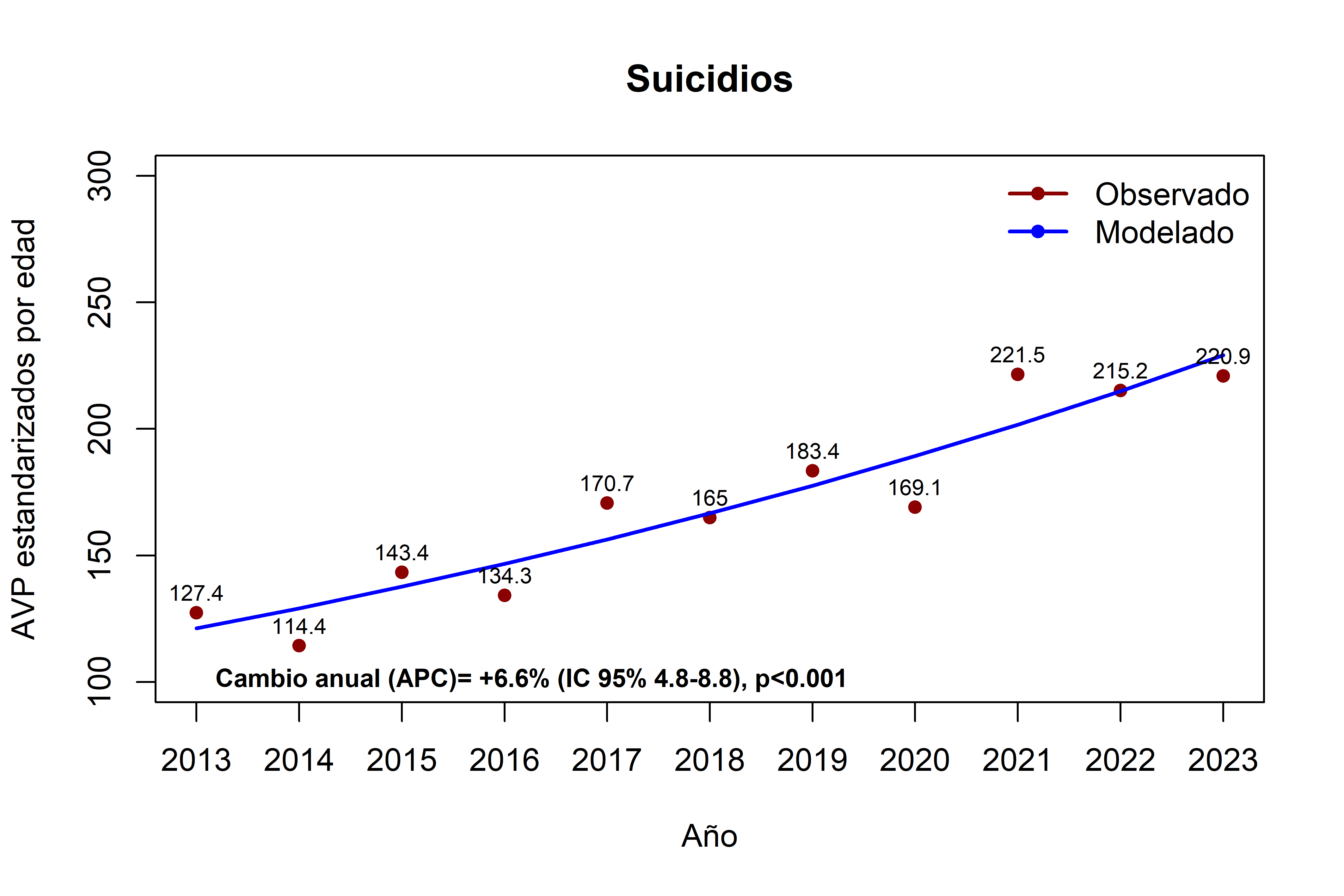


*APC= Porcentaje de cambio anual

**Figura 2. Joinpoint regression de años de vida perdidos estandarizados por edad por 100,000 habitantes en causas externas en mujeres, Honduras, 2013-2023**


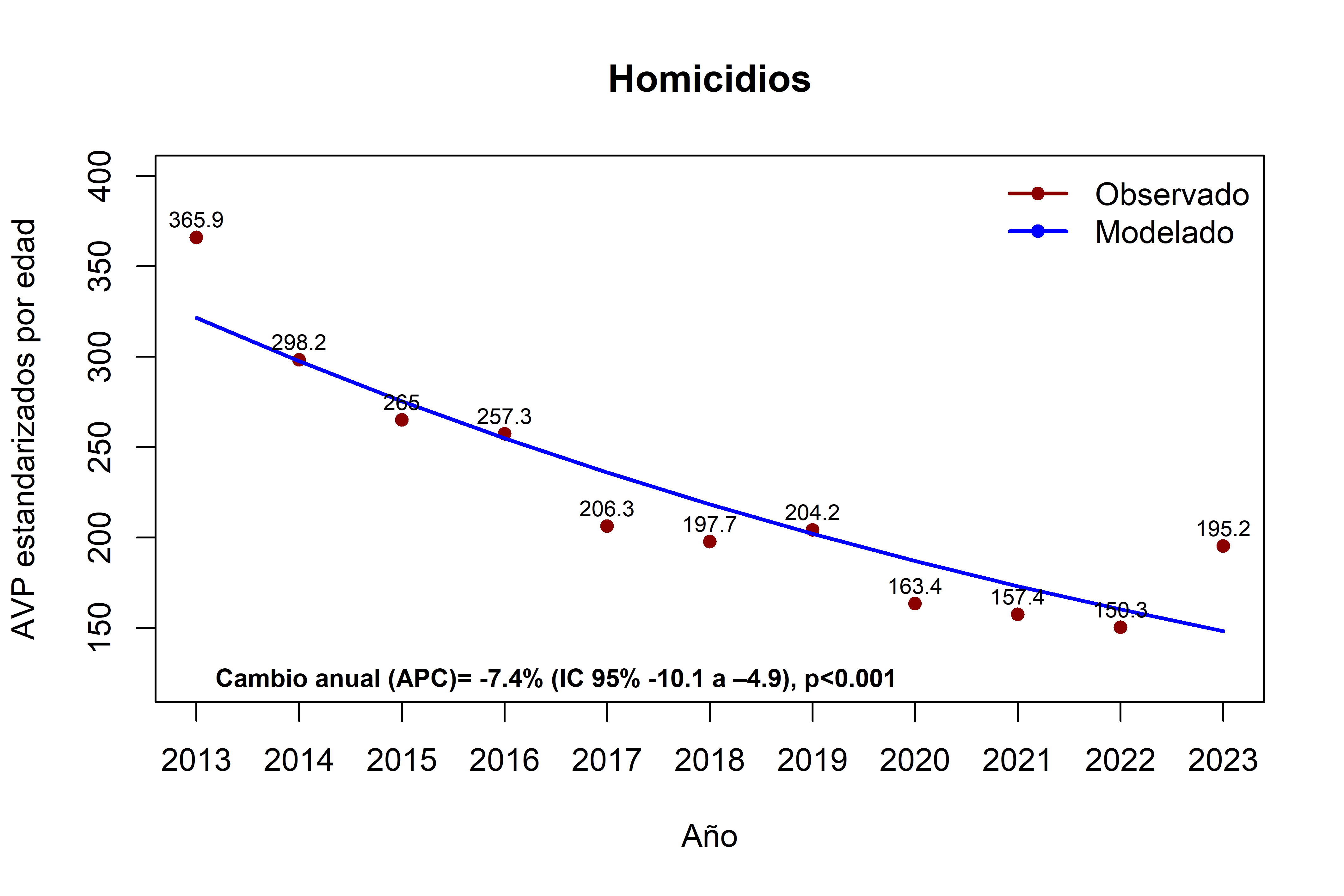

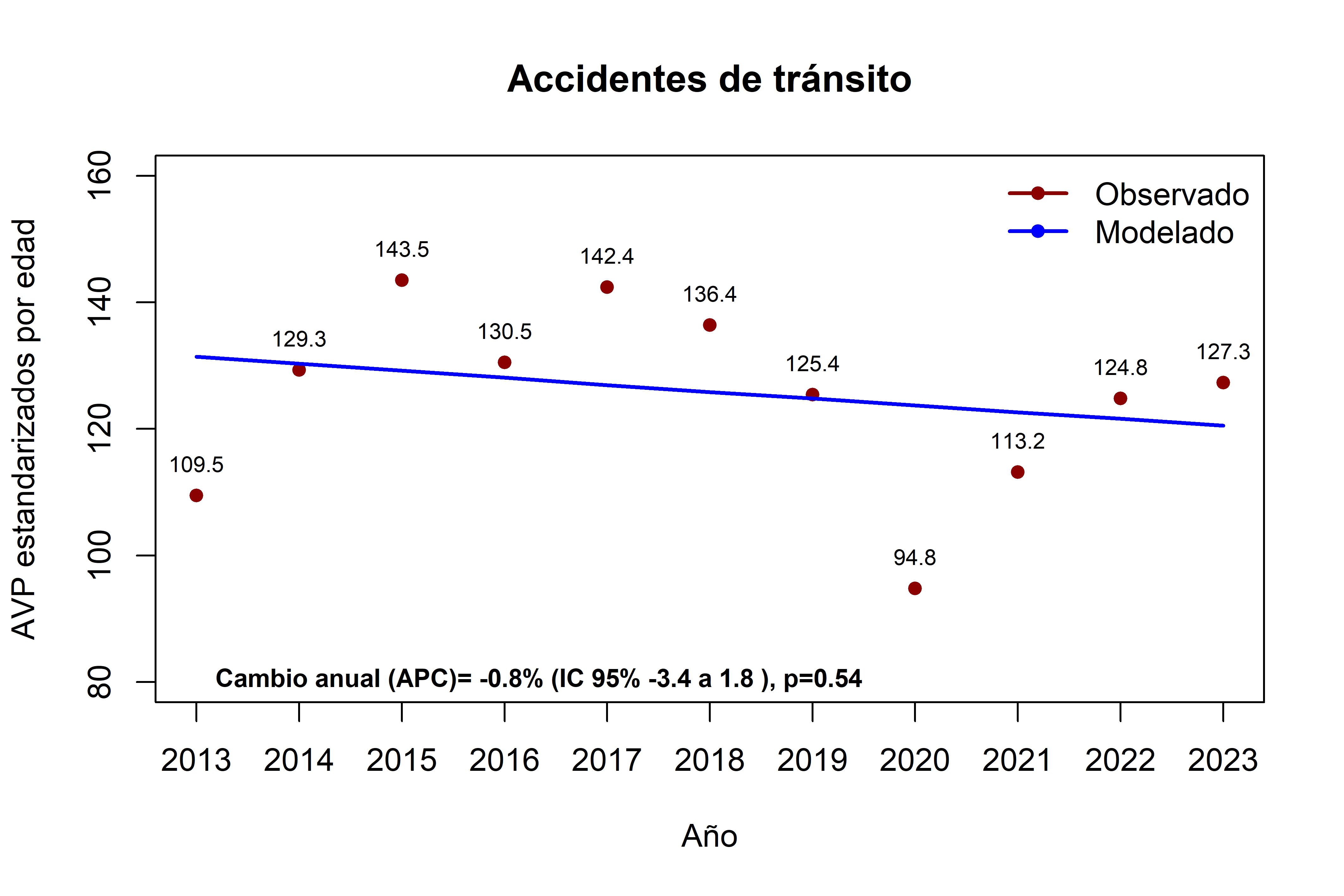

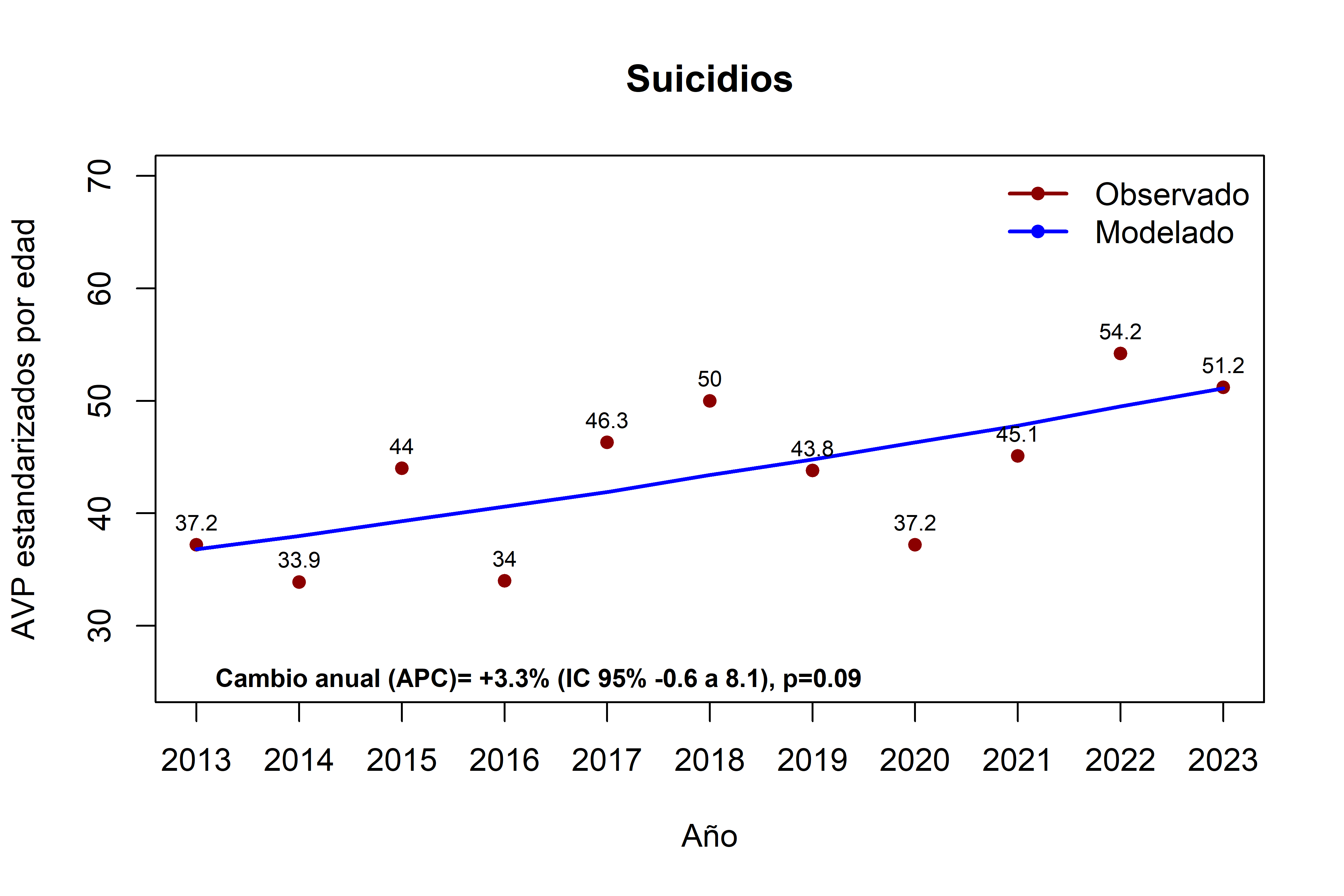

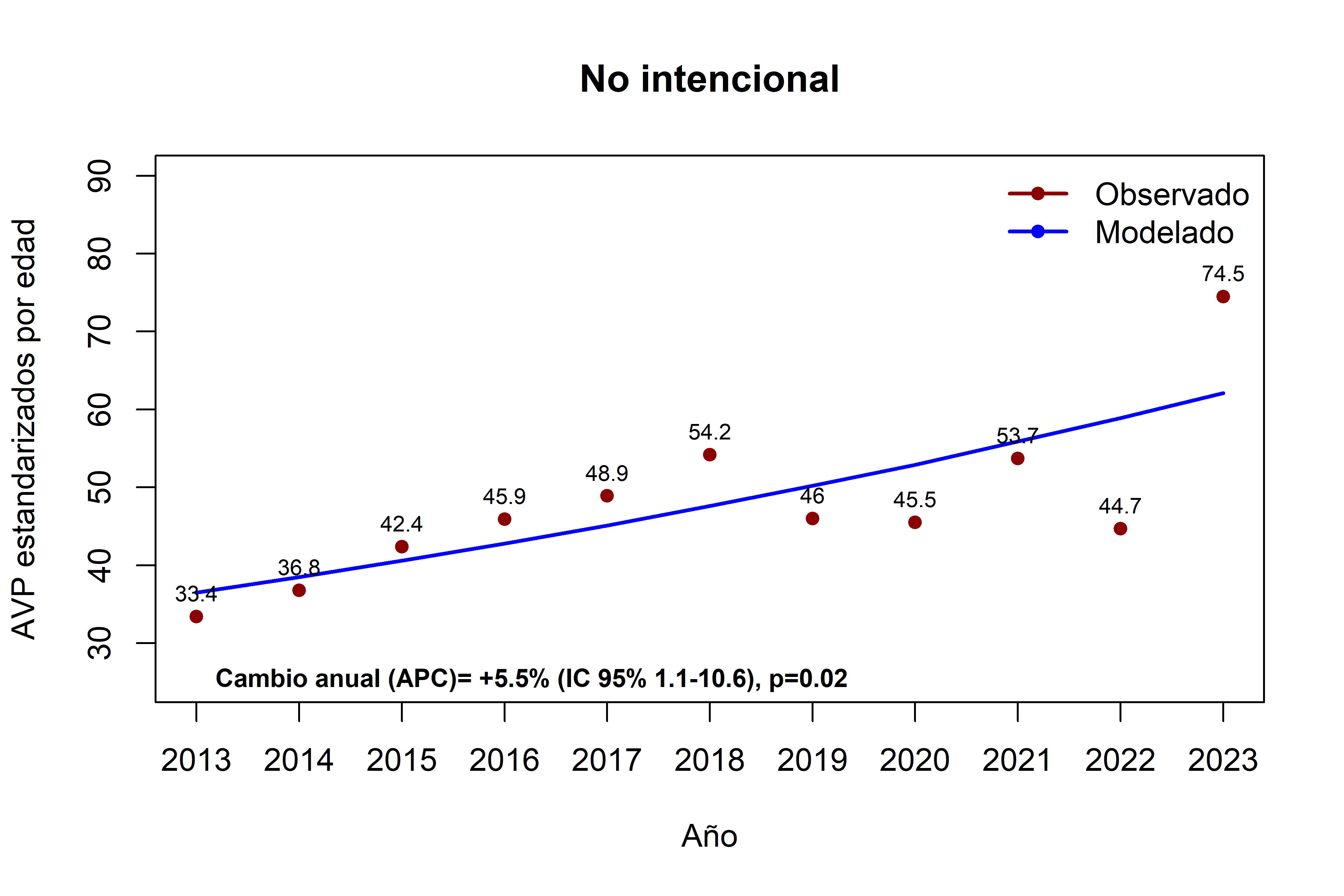


*APC= Porcentaje de cambio anual
