# Supplementary material for: Years of life lost due to external causes in Honduras, 2013-2023
Source: Rev Peru Med Exp Salud Publica. 2026 Mar 31;43(1):69–76. doi: 10.17843/rpmesp.2026.431.15420 (PMC13245990; doi:10.17843/rpmesp.2026.431.15420)
Supplement: Translation

# Translation: Años de vida perdidos por causas externas en Honduras, 2013-2023

**Authors:** Augusto A. Rosales-Meléndez

**Affiliations:** 1 Investigador independiente; Tegucigalpa, Honduras.

**Keywords:** Esperanza de Vida, Mortalidad, Causas Externas, Honduras, Mortalidad Prematura

## Abstract

**Objetivos.:**

Estimar los años de vida perdidos (AVP) para las muertes por causa externa en Honduras durante el periodo 2013-2023.

**Materiales y métodos.:**

Estudio descriptivo-ecológico, basado en registros de mortalidad por causas externas del Observatorio Nacional de la Violencia de Honduras (2013-2023). Se estimaron los AVP y se realizó un análisis estratificado por sexo, edad y tipo de causa. Adicionalmente, se efectuó un análisis de tendencias mediante la prueba de Mann-Kendall, estratificado por sexo y tipo de causa.

**Resultados.:**

Durante el período analizado se estimaron 1,9 millones de AVP por causas externas, de los cuales el 86% correspondió a hombres. Los homicidios fueron la principal causa de AVP en ambos sexos (62,4% en hombres y 43,5% en mujeres), seguidos por los accidentes de tránsito (18,3% y 25%, respectivamente). Entre 2013 y 2023, los AVP mostraron un aumento sostenido. En hombres, los incrementos se observaron en causas no intencionales (110,5%), suicidio (100,1%) y accidentes de tránsito (98,7%); en mujeres, en causas no intencionales (146,2%), suicidio (62,6%) y accidentes de tránsito (40,4%).

**Conclusiones.:**

Las muertes por causas externas, en su mayoría prevenibles, afectan de forma desproporcionada a la población adulta joven y muestran una tendencia creciente. Es fundamental implementar intervenciones diferenciadas por edad y sexo que contribuyan a reducir su impacto en la mortalidad prematura.

## INTRODUCCIÓN

Las muertes por causas externas incluyen homicidios, accidentes de tránsito, suicidios y otras lesiones no intencionales como caídas, electrocución y accidentes laborales, representan una de las principales causas de mortalidad, tanto en países de altos ^1^ y de bajos ingresos.^2^. Solo en la región de las Américas para el año 2019 se estimó la pérdida de 38 millones de años de vida perdidos (AVP) por muertes de causa externa ^3^.

En el caso de Honduras, para el 2021 las muertes por causa externa representaban la segunda causa de carga de enfermedad después de las enfermedades cardiovasculares ^4^. Estas muertes afectan de manera desproporcionada a hombres y a personas jóvenes en edad productiva, lo que genera pérdidas significativas no solo en términos de salud, sino también en capital humano, productividad y bienestar familiar.

Tomando en cuenta la magnitud de las muertes por causas externas, resulta esencial no solo describir su frecuencia, sino también realizar una adecuada estratificación epidemiológica por sexo, grupo etario y tipo de causa externa. Este enfoque permite visibilizar con mayor precisión a los grupos más afectados y orientar el diseño de políticas públicas específicas y focalizadas según la edad y el sexo.

En este contexto, el uso de métricas más avanzadas de carga de enfermedad como los AVP, y su desagregación por sexo, edad y causa, aporta información crítica para la toma de decisiones en salud pública, por lo tanto, el objetivo de este estudio fue estimar la carga de enfermedad medida en AVP por muertes por causas externas en Honduras durante el período 2013-2023.

**MENSAJE CLAVE**

Motivación para realizar el estudio. Las muertes por causas externas generan una alta mortalidad en Honduras, sin embargo, su impacto pocas veces se cuantifica usando años de vida perdidos (AVP).

Principales hallazgos. Entre 2013 y 2023 se perdieron 1.9 millones de años de vida por causas externas. En ambos sexos, las principales causas fueron homicidios, accidentes de tránsito, suicidios y muertes no intencionales. La mayor carga se concentró en adultos menores de 30 años.

Implicaciones.Se requieren intervenciones diferenciadas por edad y sexo que prevengan la violencia homicida, accidentes de tránsito y suicidios.

## MATERIALES Y MÉTODOS

### Diseño de estudio

Se realizó un estudio descriptivo-ecológico mediante análisis de datos secundarios de muertes por causas externas en Honduras durante el periodo 2013-2023. La información fue obtenida del Observatorio Nacional de la Violencia (ONV), un organismo que recopila y consolida registros de mortalidad provenientes de la Policía Nacional (PN), la Dirección de Medicina Forense (DMF) y el Registro Nacional de las Personas (RNP), mediante descarga de bases de datos abiertas y anonimizadas ^5^. Adicionalmente, para realizar un análisis estadístico más detallado, se solicitó al ONV, mediante correo electrónico, acceso a registros desagregados

### Definición de variables

La clasificación de las causas externas fue realizada por el ONV y se utilizó según las categorías registradas por dicha institución. El sexo se categorizó como mujer y hombre. Las causas externas se clasificaron en homicidio, suicidio, accidentes de tránsito, causas no intencionales (caídas, quemaduras, electrocución, intoxicaciones, soterramientos y explosiones) y causas externas no consignadas, correspondientes a mecanismos de muerte no determinados. La edad se estratifico en quinquenios con el fin de mantener coherencia con los requerimientos metodológicos para la estandarización por edad y como requisito para el cálculo de los AVP.

### Análisis estadístico

Se siguió la metodología del Global Burden Disease (GBD) ^6^ para el cálculo de los AVP, primero se calculó la esperanza de vida estándar haciendo uso de las tablas de vida de la ONU ^7^ para Latinoamérica, se asignaron los valores correspondientes según sexo para la esperanza de vida al nacer, usando como referencia las estimaciones del Banco Mundial ^8^. Posteriormente para cada grupo etario, se interpoló la esperanza de vida estándar, tomando como base la edad promedio del fallecimiento. El cálculo de la esperanza de vida estándar, se realizó mediante la ecuación 1 ^6^ (material suplementario).

Posteriormente se calcularon los AVP mediante la multiplicación del número de muertes, estratificados por edad, sexo y causa externa, por los años de vida esperados según la esperanza de vida estándar. Para cada grupo etario se utilizó la edad promedio del fallecimiento y la esperanza de vida estándar interpolada correspondiente, mediante la ecuación 2 (material suplementario).

Los parámetros fueron conservadores según recomendaciones del GBD, ^6^ entre los cuales están: No aplicar descuento por edad, emplear una constante de ponderación por edad de 0,04 y un ajuste por edad de 0,165.

Se estimaron además otras medidas derivadas como el AVP promedio, calculado como el total de AVP dividido entre el número de muertes, estratificadas por sexo, edad y causa. De forma complementaria, se estimó la razón de AVP de hombres y mujeres, utilizando a los hombres como categorías de referencia.

Para el análisis de tendencia se utilizó la prueba de Mann-Kendall junto con el valor de p asociado y la pendiente estimada mediante el método de Sen. De forma complementaria, se realizó una regresión Joinpoint para identificar posibles puntos de cambio en las tendencias de los AVP, considerando el periodo de la pandemia por COVID-19 (2020-2023) como evento de interés. Para este análisis se calcularon tasas de AVP estandarizadas por edad por 100 000 habitantes, utilizando las proyecciones poblacionales del Instituto Nacional de Estadística de Honduras (INE) y la población estándar mundial de la Organización Mundial de la Salud (OMS) como ponderación. La regresión Joinpoint se realizó utilizando el software Joinpoint Trend Analysis Program, versión 5.4.0.

Los resultados se presentaron mediante gráficos de tendencia y tablas de frecuencias absolutas y relativas. Los análisis estadísticos se realizaron en RStudio (versión 2024.12.1; RStudio Team, Boston, MA, USA), usando los paquetes “dplyr” y “tidyr” para la organización de los datos, el paquete “yll” para el cálculo de los AVP y para el análisis de tendencias el paquete “trend”.

### Consideraciones éticas

Este estudio se realizó conforme a los principios éticos establecidos en la Declaración de Helsinki para la investigación médica. Se garantizó la confidencialidad mediante el uso exclusivo de datos secundarios completamente anonimizados. Dado que no se incluyó información identificable ni se involucró contacto con personas y es un análisis de datos secundarios de datos abiertos, no se requirió la aprobación de un comité de ética, conforme a lo establecido en las International Ethical Guidelines for Epidemiological Studies ^9^.

## RESULTADOS

Durante el período 2013-2023 se registraron 74724 muertes por causas externas en hombres, de las cuales 1753 (2,3%) fueron excluidas por no contar con información sobre la edad. En mujeres se reportaron 11095 muertes, excluyéndose 190 (1,7%) por la misma razón. Con los datos disponibles, se estimó una pérdida total de 1,9 millones de AVP en Honduras, de los cuales el 86% (1,64 millones) correspondió a los hombres (tabla 1).

**Tabla 1:** Años de vida perdidos (AVP) por sexo y causa, Honduras 2013-2023.

	Mujeres				Hombres				
	Muertes	AVP	%	AVP Promedio	Muertes	AVP	%	AVP Promedio	Razón* H/M
Accidentes de transito	2695	61845,5	24,1	22,9	13823	300353,8	18,3	21,7	5:1
Homicidios	4586	111682,0	43,5	24,3	43640	1024040,7	62,4	23,5	9:1
No consignada	1467	34359,0	13,4	23,5	5256	105775,4	6,4	20,1	3:1
No intencional	1233	24920,5	9,7	20,2	6531	134689,1	8,2	20,6	5:1
Suicidio	924	23737,4	9,3	25,8	3541	77516,2	4,7	21,9	3:1
Total	10905	256544,4	100	22,5	72791	1642375,2	100	23,5	6:1

* Razón AVP de Hombre: Mujer.

En ambos sexos, la principal causa de AVP fueron los homicidios. En hombres, se perdieron 1 millón de AVP (62,4%), mientras que en mujeres fueron 111 mil AVP (43,5%), con una razón hombre: mujer de AVP de 9:1. La segunda causa fueron los accidentes de tránsito, con 300 mil AVP en hombres (18,3%) y 61 mil en mujeres (25%) (tabla 1).

En otras causas como las muertes no intencionales, el suicidio y las causas externas no consignadas, se observaron razones hombre: mujeres igualmente elevadas: 5:1, 6:1 y 3:1, respectivamente. Cabe destacar que, en términos relativos, las mujeres registraron 34 mil AVP por causas externas no especificadas (13,4%), mientras que los hombres acumularon 100 mil AVP (6,4%). En el caso del suicidio, las mujeres perdieron 23 mil AVP (9,3%) y los hombres 70 mil AVP (4,7%) (tabla 1).

Durante el período 2013-2023, el análisis de tendencias mediante la prueba de Mann-Kendall y la pendiente de Sen mostró tendencias crecientes estadísticamente significativas en los AVP por causas no intencionales, suicidio y accidentes de tránsito en ambos sexos. La única excepción correspondió a los accidentes de tránsito en mujeres, en los que no se identificó una tendencia estadísticamente significativa. En contraste, los homicidios presentaron tendencias decrecientes tanto en hombres como en mujeres (tabla 2).

**Tabla 2:** Tendencia temporal y pendientes por causas externas en Honduras 2013-2023.

Causa externa	Hombres			Mujeres		
	Tau^a^	p^b^	Pendiente^c^	Tau^a^	p^b^	Pendiente^c^
No intencional	0,78	0,001	544 (326,2 a 900,8)	0,75	0,001	131 (39,4 a 236,1)
Suicidio	0,85	<0,001	559 (457,5 a 721,8)	0,60	0,01	104 (45,1 a 179,3)
Tránsito	0,81	<0,001	1810 (1331,8 a 2324,6)	0,27	0,27	75 (-75,6 a 262,0)
Homicidio	-0,81	<0,001	−6290 (-9172,3 a -2807,4)	-0,75	0,001	-648 (-892,7 a -364,7)

a Tau de Mann Kendall, ^b^ Valor de p de Mann Kendall, ^c^ Valor de la pendiente en años de vida perdidos (Método Sen).

Desde el 2013 al 2023, los AVP en hombres se incrementaron en un 110,5% para causas no intencionales, 100,1% para suicidios y 98,7% para accidentes de tránsito, mientras que los homicidios disminuyeron un 52,2% (figura 1). En las mujeres se observó un patrón similar, con incrementos del 146,2% en causas no intencionales, 62,6% en suicidios y 40,4% en accidentes de tránsito, y una reducción del 37,3% en los AVP por homicidio (figura 2). En ambos sexos se observó un descenso de los AVP por accidentes de tránsito en el año 2020.

**Figura 1:** Anos de vida perdidos por causas externas en hombres, Honduras, 2013-2023.

**Figura 2:** Años de vida perdidos por causas externas en mujeres, Honduras, 2013-2023.

En los hombres, los menores de 30 años concentraron el 51,0% de los AVP acumulados por accidentes de tránsito, el 53,9% por homicidios, el 49,3% por suicidios y el 44,6% por causas no intencionales (tabla 3). Asimismo, al considerar las mujeres menores de 30 años, se acumuló el 53,6% de los AVP por accidentes de tránsito, el 54,7% de los homicidios, el 73,9% de los suicidios y el 61,6% de las causas no intencionales. Destaca el grupo de 15 a 19 años, que representó el 31% de los AVP por suicidio en mujeres, así como el grupo de 25 a 29 años, que aportó el mayor porcentaje individual para las cuatro causas descritas (tabla 4). El análisis de tendencia mediante regresión Joinpoint no identificó puntos de cambio estadísticamente significativos en las tendencias de los AVP para ninguno de los tipos de causas externas analizados en ambos sexos, lo que indica la presencia de una única tendencia temporal durante todo el periodo de estudio, incluidos los años de la pandemia por COVID-19 (material suplementario).

**Tabla 3:** Años de vida perdidos según edad y causa externa en hombres, Honduras, 2013-2023.

Edad	Accidentes de tránsito n (%)	Porcentaje acumulado^a^	Homicidios n (%)	Porcentaje acumulado^a^	Suicidio n (%)	Porcentaje acumulado^a^	No consignada n (%)	Porcentaje acumulado^a^	No intencional n (%)	Porcentaje acumulado^a^
0-4	5272 (1,8)	1,8	5298,7 (0,5)	0,5	-	-	14737,1 (13,9)	13,9	8421,1 (6,3)	6,3
5-9	6601,2 (2,2)	4,0	2191,2 (0,2)	0,7	143 (0,2)	0,2	1150,4 (1,1)	15	5101 (3,8)	10,1
10-14	8233,5 (2,7)	6,7	9926,3 (1)	1,7	1850,6 (2,4)	2,6	1659,2 (1,6)	16,6	5622,1 (4,2)	14,3
15-19	28624,7 (9,5)	16,2	116563,7 (11,3)	13,0	8672,6 (11,2)	13,8	4312,2 (4,1)	20,7	12180,4 (9)	23,3
20-24	56260,5 (18,7)	35,0	216129,2 (21,1)	34,1	14721,9 (19)	32,8	7944,7 (7,5)	28,2	14075,1 (10,5)	33,8
25-29	48109,4 (16)	51,0	202710,1 (19,8)	53,9	12851,7 (16,5)	49,3	9376,9 (8,9)	37,1	14530 (10,8)	44,6
30-34	36999,8 (12,3)	63,4	154508,4 (15,1)	69,0	8864,1 (11,3)	60,6	12100 (11,3)	48,4	13456,5 (10)	54,6
35-39	28804,7 (9,7)	73,0	112568,5 (11)	80,0	8101,5 (10,5)	71,1	10766,1 (10,2)	58,6	13100,9 (9,7)	64,3
40-44	19717,2 (6,6)	79,6	76630,5 (7,5)	87,5	6839,3 (8,8)	79,9	9394,9 (8,9)	67,5	11442,3 (8,5)	72,8
45-49	15944,1 (5,3)	84,9	50734,5 (5)	92,5	5079,2 (6,6)	86,5	8,643,5 (8,2)	75,7	9340,2 (6,9)	79,7
50-54	13357,9 (4,4)	89,3	31819,2 (3,1)	95,6	3221,4 (4,2)	90,7	6,948,2 (6,6)	82,3	7948,5 (5,9)	85,6
55-59	10359,1 (3,4)	92,7	21897,9 (2,1)	97,7	2473,9 (3,2)	93,9	6,502,8 (6,1)	88,4	5826,9 (4,3)	89,9
60-64	8118 (2,7)	95,4	12047 (1,2)	98,9	1797,6 (2,3)	96,2	4,133,0 (3,9)	92,3	4904,1 (3,6)	93,5
65-69	6093,6 (2,0)	97,4	6249,8 (0,6)	99,5	1235,1 (1,6)	97,8	3,644,6 (3,4)	95,7	3473,5 (2,6)	96,1
70-74	3562,5 (1,2)	98,6	2855,1 (0,3)	99,8	755,9 (1)	98,8	2,180,6 (2,1)	97,8	2184 (1,6)	97,7
75-79	2207,1 (0,7)	99,3	1228,5 (0,1)	99,9	509,5 (0,7)	99,5	1,346,4 (1,3)	99,1	1397,7 (1)	98,7
80-84	2088,6 (0,7)	100,0	682 (0,1)	100,0	399 (0,5)	100	934,7 (0,9)	100	1684,8 (1,3)	100
Total	300353,8 (100)	-	1024040,7	-	77516,2 (100)	-	105775,4	-	134689,1 (100)	-

a Porcentaje acumulado según causa y edad.

Nota: Los valores entre paréntesis representan el porcentaje respecto al total de años de vida perdidos según causa y edad.

**Tabla 4:** Años de vida perdidos según edad y causa externa en mujeres, Honduras, 2013-2023.

Edad	Accidentes de tránsito n (%)	Porcentaje acumulado^a^	Homicidios n (%)	Porcentaje acumulado^a^	Suicidio n (%)	Porcentaje acumulado^a^	No consignada n (%)	Porcentaje acumulado^a^	No intencional n (%)	Porcentaje acumulado^a^
0-4	4490,3 (7,3)	7,3	3390,2 (3,0)	3,0	-	-	10681,1 (30,9)	30,9	6188,3 (24,8)	24,8
5-9	4061,7 (6,6)	13,8	969,5 (0,9)	3,9	29,3 (0,1)	0,1	1000,4 (2,9)	2,9	2442,1 (9,8)	34,6
10-14	3510,3 (5,7)	19,5	3930,4 (3,5)	7,4	2527,6 (10,5)	10,6	1839,1 (5,3)	5,3	2074,5 (8,3)	42,9
15-19	6232,3 (10,1)	29,6	16225 (14,5)	21,9	7371 (31,1)	41,7	2749,4 (8,0)	8,0	1686,7 (6,8)	49,7
20-24	7998,7 (12,9)	42,5	18821 (16,8)	38,7	4503,3 (19,0)	60,7	2892,6 (8,4)	8,4	1583,3 (6,4)	56,1
25-29	6862,3 (11,1)	53,6	17855,1 (16)	54,7	3139 (13,2)	73,9	2424,4 (7)	7,0	1368,7 (5,5)	61,6
30-34	6392,4 (10,3)	63,9	13484,6 (12,1)	66,8	1466,6 (6,2)	80,1	2369,2 (6,9)	6,9	1214,6 (4,9)	66,5
35-39	4819 (7,8	71,7	11714,3 (10,5)	77,3	1490,6 (6,3)	86,4	2501,3 (7,2)	7,2	983,7 (3,9)	70,4
40-44	3696,8 (6,0)	77,7	8448,6 (7,6)	84,9	1083,7 (4,6)	91,0	1970,8 (5,7)	5,7	924,9 (3,7)	74,1
45-49	3229,7 (5,2)	82,9	6386,7 (5,7)	90,6	800,5 (3,4)	94,4	1318,8 (3,8)	3,8	1179,5 (4,7)	78,8
50-54	2801 (4,5)	87,5	4329,8 (3,9)	94,5	501,4 (2,1)	96,5	1042,7 (3)	3,0	814,8 (3,3)	82,1
55-59	2287 (3,7)	91,2	2560,9 (2,3)	96,8	435,8 (1,8)	98,3	1150,3 (3,3)	3,3	482,8 (1,9)	84,0
60-64	2004,8 (3,2)	94,4	1656,7 (1,5)	98,3	151 (0,6)	98,9	591,8 (1,7)	1,7	762,3 (3,1)	87,1
65-69	1603,7 (2,6)	97,0	879,9 (0,8)	99,1	93,9 (0,4)	99,3	745,5 (2,2)	2,2	493,9 (2,0)	89,1
70-74	879,8 (1,4)	98,4	481,9 (0,4)	99,5	75,9 (0,3)	99,6	505,2 (1,5)	1,5	639,1 (2,6)	91,7
75-79	508,6 (0,8)	99,2	314,1 (0,3)	99,8	61,5 (0,3)	99,9	298,1 (0,9)	0,9	631,4 (2,5)	94,2
80-84	467,2 (0,8)	100,0	233,2 (0,2)	100,0	6,2 (0,1)	100	458,9 (1,3)	1,3	1450 (5,8)	100
Total	61845,5 (100)	-	111682 (100)	-	23737,4 (100)	-	34539,6 (100)	100,0	24920,5 (100)	-

a Porcentaje acumulado según causa y edad.

Nota: Los valores entre paréntesis representan el porcentaje respecto al total de años de vida perdidos según causa y edad.

## DISCUSIÓN

Entre 2013 y 2023, la evolución de los AVP por causas externas en Honduras mostró cambios en el perfil de la mortalidad. Si bien los homicidios continuaron siendo la causa predominante, su peso relativo disminuyó de manera considerable en ambos sexos. En contraste, los AVP asociados a accidentes de tránsito, suicidio y causas no intencionales experimentaron un crecimiento sostenido. Este patrón sugiere avances en el control de la violencia homicida, pero también evidencia un aumento en las demás muertes por causas externas que requieren atención urgente desde la salud pública.

Más allá de los cambios en el perfil de la mortalidad por causas externas, persiste una asimetría marcada por sexo en la mortalidad por homicidios, con un predominio consistente de los hombres en todos los grupos etarios. Este patrón ha sido ampliamente documentado tanto en países cercanos cultural y geográficamente, como México ^10^ y también en contextos más distantes, como Irán ^11^.

Una posible explicación para la desigualdad por sexo ha sido atribuida a una mayor participación masculina tanto como perpetradores como víctimas de la violencia. Se estima que aproximadamente 9 de cada 10 delitos violentos son cometidos por hombres ^12^ y el 81% de las víctimas también son hombres ^13^. Esta doble condición ha sido vinculada a factores estructurales como la participación en redes del crimen organizado y la exposición a violencia interpersonal, especialmente entre jóvenes de 15 a 29 años ^13^. También se ha señalado una mayor tendencia entre los hombres a resolver conflictos mediante el uso de la fuerza ^14^, así como la influencia de normas de masculinidad que refuerzan la violencia como forma de validación social ^15^. Estos datos respaldan la necesidad de políticas focalizadas en hombres jóvenes en situación de riesgo.

Los accidentes de tránsito constituyeron la segunda causa de AVP, con una mayor carga absoluta en los hombres. Estas diferencias por sexo son consistentes con estudios previos. En consonancia con ello, en Irán se ha reportado que los hombres presentan más del triple de AVP por esta causa en comparación con las mujeres ^16^, mientras que en México también se ha observado una predominancia masculina en todas las edades en la mortalidad por accidentes de tránsito ^17^.

Desde un enfoque etiológico, estas diferencias por sexo en la carga de AVP por accidentes de tránsito pueden explicarse, en parte, por factores como el predominio masculino en el rol de conductor de motocicleta, un medio de transporte asociado a alta letalidad ^18^. Asimismo, se ha descrito una mayor tendencia entre los hombres a incurrir en conductas imprudentes y a incumplir las normas de tránsito, tanto como conductores ^19^ como peatones ^20^. El incremento de los AVP por accidentes de tránsito durante el período 2013-2023 podría estar asociado al crecimiento del parque vehicular, incremento de la densidad vial y a un mayor riesgo de siniestralidad en el entorno urbano ^(^^21^. En 2020 se registró una interrupción puntual de la tendencia, con un descenso de los AVP en ambos sexos, probablemente asociado a las medidas de aislamiento implementadas durante la pandemia del COVID-19 y a la consiguiente reducción de la movilidad vehicular; un comportamiento similar en la mortalidad fue reportado en otros países que adoptaron medidas de contención análogas ^22^.

En cuanto al suicidio, el análisis de los AVP también encontró un predominio marcado en los hombres, no obstante, la carga relativa fue dos veces mayor en las mujeres (9,3% frente a 4,7%), influenciada principalmente por la alta frecuencia de suicidios en edades más tempranas. En este estudio, uno de cada tres AVP por suicidio en mujeres correspondió al grupo de 15 a 19 años. Este patrón es consistente con series epidemiológicas internacionales que reportan una elevada incidencia de suicidio en mujeres jóvenes ^23^. El incremento de los AVP asociados al suicidio podría estar relacionado con la presencia de trastornos mentales no tratados, condiciones sociodemográficas vulnerables y la exposición a eventos adversos a lo largo de la vida ^24^. Estos resultados subrayan la necesidad de fortalecer los programas de salud mental, con especial énfasis en mujeres jóvenes y hombres en edad productiva.

De forma paralela, los AVP por causas no intencionales (como caídas, electrocución y accidentes laborales), presentaron un predominio marcado en los hombres, quienes registraron cinco veces más AVP por esta causa en comparación con las mujeres. Este patrón ha sido ampliamente documentado en la literatura, y se presenta de forma consistente en todos los grupos etarios, igualándose únicamente en edades avanzadas, a partir de los 80 años ^25^.

Esta desigualdad puede ser atribuida a una mayor exposición de los hombres a riesgos laborales que incrementan la probabilidad de accidentes fatales, como trabajos físicamente exigentes o desarrollados bajo condiciones ambientales adversas (calor extremo, humedad, exposición a gases o sustancias tóxicas). En contraste, las mujeres tienden a estar más expuestas a formas de violencia psicológica en el entorno laboral ^(^^26^. Además, el aumento de los AVP por causas no intencionales podría estar vinculado tanto a una mejora en los sistemas de notificación como a condiciones de trabajo precarias, el incumplimiento de medidas de seguridad ocupacional y la prolongación de las jornadas laborales ^27^.

En cuanto a la distribución por edad, los menores de 30 años representaron la mayor contribución a los AVP totales en ambos sexos. Desde una perspectiva macroeconómica, la alta mortalidad en este grupo etario genera consecuencias económicas como la reducción de la fuerza laboral y las pérdidas de ingreso. Se estima que, solo por accidentes de tránsito, la región de las Américas pierde entre un 3% y un 5% de su producto interno bruto (PIB) cada año, lo que refleja la magnitud de esta carga evitable sobre el desarrollo económico y social ^28^.

Este estudio no está exento de limitaciones. Entre ellas se encuentran la exclusión de aproximadamente el 2% de los registros debido a la falta de información sobre la edad, lo que impidió el cálculo correspondiente de los AVP. Asimismo, existe la posibilidad de sesgos debido a errores en la calidad de los datos, la clasificación de causas o el subregistro de muertes. Debe considerarse también la ausencia de variables socioeconómicas o contextuales, así como los efectos diferidos de fenómenos como la pandemia por COVID-19, que pudieron influir en la mortalidad.

No obstante, entre las fortalezas se destaca una muestra representativa y de más de 10 años, que permite describir de forma representativa la carga de mortalidad por causas externas en el país. Además, se emplearon metodologías estandarizadas para el cálculo de AVP, lo que favorece la comparabilidad y consistencia de los resultados.

En conclusión, las muertes por causas externas generan una elevada carga para el país, especialmente en hombres y adultos jóvenes menores de 30 años. La acumulación de una carga de AVP seis veces mayor en hombres que en mujeres refuerza la necesidad de incorporar enfoques de género en las políticas públicas, así como de diseñar intervenciones focalizadas que contribuyan a reducir la mortalidad prematura en los grupos más vulnerables.

Si bien se encontró una disminución en los AVP por homicidio en ambos sexos, esta causa continúa siendo la principal entre las muertes externas, lo que subraya la importancia de mantener y fortalecer las estrategias de prevención de la violencia y la criminalidad. Por otro lado, el aumento de los AVP por accidentes de tránsito, suicidio y causas no intencionales en ambos sexos pone en evidencia la urgencia de implementar acciones diferenciadas por grupo etario y género, que aborden de manera integral la seguridad vial, la salud mental y las condiciones de seguridad en los entornos laborales y comunitarios.
